# Relationship Between Brand Presence and Emotions on Overall Acceptance and Purchase Intent of Commercial Chicken Noodle Soup

**DOI:** 10.3390/foods14203505

**Published:** 2025-10-15

**Authors:** Derui Wendell Loh, Adam Parker, Laura Jefferies

**Affiliations:** 1 Department of Nutrition, Dietetics & Food Science, Brigham Young University, S133 Eyring Science Center, Provo, UT 84602, USA; wendellloh@gmail.com; 2 JSA Nutrition, 138 E 12300 S #282, Draper, UT 84020, USA; adam@jsanutrition.com

**Keywords:** emotions, brand presence, sensory acceptance, purchase intent, comfort food, chicken noodle soup

## Abstract

This study examined the influence of brand presence and discrete emotions on consumer acceptance and purchase intent of commercial chicken noodle soups. A total of 324 evaluations across three soup categories (chunky, low-sodium, condensed) were conducted under blind and unblinded conditions using a 42-term emotion lexicon. Brand presence did not exert moderate-to-large effects, though subtle brand-specific differences cannot be excluded. Instead, three emotions, “satisfied,” “disgusted,” and, for condensed soups, “bored,” emerged as the strongest predictors, together explaining a substantial proportion of variance in liking and purchase intent. Many other positive emotions clustered around “satisfied,” highlighting a parsimonious set of dominant drivers. Quiet positive emotions such as contentment, peacefulness, and warmth consistently aligned with both acceptance and purchase intent. These findings extend prior research by showing that consumer responses consolidate around a limited set of emotions, underscoring that evoking subtle, self-focused positive feelings may be more effective in comfort food marketing and product development than reliance on brand identity or nostalgia.

## 1. Introduction

Brand information is a powerful marketing tool that can shape consumer choices. Prior studies demonstrate that brand familiarity influences perceptions of quality, acceptance, and purchase intent across various food products [1,2,3,4,5,6,7,8]. At the same time, emotions strongly affect food choice, consumption, and enjoyment, with both positive and negative feelings guiding consumer behavior [9,10,11,12,13].

Research suggests that brand cues may enhance liking and willingness to purchase via emotional pathways. For instance, brand information altered hedonic responses to pasta and beer [7,8]. while packaging influenced preferences for olive oils [14]. Similarly, emotions elicited during eating, such as satisfaction, contentment, or disgust—are closely tied to acceptance and purchase intent [15,16,17].

Various models have been developed to classify food-related emotions, including Ekman’s discrete theory [18], Russell’s circumplex model [19], and the HUMAINE framework [20]. Tools such as the Geneva Emotion and Odor Scale [21], the EsSence lexicon [18], and subsequent food-specific emotion lexicons [22,23] provide structured approaches for capturing consumer emotions.

Despite this progress, few studies have isolated the independent influence of brand presence on both emotions and purchasing, particularly for staple comfort foods. Understanding whether brand cues add value beyond intrinsic product qualities is relevant for both marketing and product development.

This study investigates how the relative influence of brand presence and discrete emotions on consumer acceptance and purchase intent for commercial chicken noodle soup. We hypothesized that (1) certain emotions would consistently align with overall acceptance, (2) brand knowledge would alter acceptance, emotions, and purchase intent, and (3) emotional profiles would vary across soup types (chunky, low-sodium, condensed).

## 2. Materials and Methods

### 2.1. Samples

Samples of commercial chicken noodle soup, with brands ranging from nationally recognized to those with private labels, were purchased from local grocery stores; one private label sample was obtained through a church-based organization serving people in need, which was generally recognized in the test area. Chicken noodle soup was selected as a model for this study because it is commonly associated with comfort during times of emotional distress or illness [24,25]. A wide range of samples was selected to represent the breadth of consumer experiences with commercial chicken noodle soup in the test area. Soups were divided into three categories: those with large pieces of chicken, vegetables and wide or spiral noodles [chunky (7 samples)], those with low-sodium, small chicken pieces and thin noodles [low-sodium (3 samples)], and those with small pieces of chicken and thin noodles [condensed (3 samples)], and were evaluated by category. The disparity between the number of soups in each category was due to market availability.

### 2.2. Sensory Evaluation

Panels were conducted at the Brigham Young University Sensory Laboratory (Provo, UT, USA). Two separate panels of 112 participants evaluated the low-sodium and condensed soups, while a separate panel of 110 participants assessed the chunky soups. The same individuals returned for both the blind and unblinded sessions within their assigned soup category, separated by two weeks, but no panelist participated in more than one soup category. This design ensured within-subject comparisons for each category while avoiding cross-category carryover effects. Panelists were recruited from a database of the campus and outlying communities and selected for their willingness to evaluate chicken noodle soup, brand familiarity, and reported eating it at least once every 3 months. Additionally, panelists for the low-sodium panels were screened for having consumed low-sodium chicken noodle soup within the past 3 months. For each panel, both genders were equally represented, with approximately equal representation across age categories from 20 to 29 years, 30 to 39 years, 40 to 49 years, 50 to 59 years, and 60 years and older. The study was approved by the University Institutional Review Board (IRB #000119), and panelists provided their informed consent. Soups were prepared, 3 or 4 cans at a time, according to the label instructions, and heated to 170 °F (76.67 °C) for 15 min on a gas stovetop in identical 3-quart stainless steel saucepans. Heated soups were then transferred to stainless steel pans and held on a steam table whose temperature cycled between 155 °F (68.33 °C) and 165 °F (73.89 °C). Fresh soups were prepared each hour.

Panelists received approximately 60 mL of each soup served into opaque plastic portion cups, in a randomized order, labeled with 3-digit blinding codes. Samples were received through pass-through compartments in isolated booths. Samples were presented sequentially and monadically in a completely randomized order. Panelists were instructed to use a bite of a cracker and a sip of bottled water to refresh their sense of taste between samples. The soup brand names were not displayed in the first panel (blind). In the second panel (unblinded), only the brand name of each soup, all in the same font and text, was revealed before evaluation.

Questions were presented one at a time on a computer screen, and data were collected using Compusense^®^5 (version 5.6) software (Compusense Inc., Guelph, ON, Canada). For each sample at both visits, panelists were asked to rate their overall acceptance of the soup on a discrete 9-point hedonic scale, with 1 indicating ‘dislike extremely’, 5 indicating ‘neither like nor dislike’, and 9 indicating ‘like extremely’. However, for the second panel, the brand name was disclosed before the questioning began. Subsequently, panelists were presented with an alphabetical list of forty-two emotions, 39 from King and Meiselman [22] and three more we chose to include—comfortable, content, and relaxed. Each emotion had an accompanying 100-point line scale on which panelists were asked to rank the intensity with which they felt each emotion on a 100-point continuous line scale labeled “not at all” on the far-left side to “extremely” on the far-right side. Finally, panelists were asked how likely/unlikely they were to purchase that soup on a discrete 9-point scale ranging from 1 = extremely unlikely, 5 = neither likely nor unlikely, to 9 = extremely likely. Panelists provided informed consent and were compensated monetarily for their time.

### 2.3. Statistical Analysis

Initially, we performed stepwise regressions of the complete model to examine the influence of each emotion on consumers’ overall acceptance of the soups and their intent to purchase the soup in the future. Independent variables included soup category and a grouping of all types, as well as blind versus unblinded presentation. Dependent variables were hedonic scores and emotion intensity. Initial full-model stepwise regression indicated that brand presence was not a significant predictor. The final reduced model revealed that the effect on overall acceptance and purchase intent was primarily driven by three emotions. A post hoc sensitivity power analysis was conducted using G*Power 3.1 to determine the minimum effect sizes that could be detected with the available sample sizes at α = 0.05 and power = 0.80. For paired blind vs. unblinded comparisons (N = 100–112), the design was sensitive to within-subject effects of dz ≈ 0.27–0.28 (Cohen’s d ≈ 0.23–0.37). For across-category analyses (N = 324), the study was sensitive to omnibus effects of f ≈ 0.174 (η^2^ ≈ 0.029). Thus, the study was adequately powered to detect small to moderate effects, although smaller effects may not have been detected.

## 3. Results and Discussion

### 3.1. Brand Presence

Brand presence had no practical positive or negative impact on panelists’ emotional responses regarding changes in overall acceptance and purchase intent for all soups combined and individual soup types. While Table 1 and Table 2 show variation between the blind and unblinded panels, the differences in the values of the incremental change in key emotions per unit change in overall acceptance were not statistically significant. The results of this study contrast those of previous studies, which concluded that brand and packaging information on both food products, such as dried semolina pasta, and non-food products could encourage positive emotions and significantly improve liking [7,13]. Such phenomena have been described as a result of “Brand Comfort”, where brands that are perceived to be capable of eliciting positive emotions are more likely to be sought out by consumers [26]. However, based on the results of this study, it is possible that brand information on certain types of food products (such as canned chicken noodle soup in this case) does not elicit a sufficient emotional response to affect overall acceptance or purchase intent. This, perhaps, could be because brand information affects certain types of food more than others due to consumer expectations surrounding different types of foods. Canned chicken noodle soup could fall into the category of “daily food”, where brand information matters less because consumers do not tend to view private label brands or major brands with much discernment or discrimination, thus explaining why brand presence did not seem to have any practical effects on panelists’ emotional responses. Although brand presence showed no overall effect, this null result may reflect a balance of opposing brand-specific responses, where stronger positive associations with nationally recognized brands were offset by less favorable responses to private labels, resulting in a net neutral outcome.

Additionally, the different types of soups did not all yield the same patterns of difference in responses between blind and unblinded panels. For the low-sodium and chunky soups, panelists expressed a greater positive change in feeling “satisfied” and a smaller change in feeling “disgusted” when the brand was revealed. In contrast, the opposite pattern was observed for condensed soups. Regardless of this disparity, the difference in relationships has no practical application. It should be noted, however, that the different emotional response profiles among the varieties of chicken noodle soups are consistent with a study by Spinelli et al. [13], who found that the emotional changes between informed and blind tasting differed depending on the hazelnut spread sample. Furthermore, while the analysis does not contrast the responses for blind and unblinded panels between popular and private label brands, the present analysis suggests that those distinct values would be similar to the reported combined values and that no additional helpful information to the marketing and study of chicken noodle soups, popular or private label, would emerge.

Although brand presence exerted no significant influence in aggregate, this null effect may reflect a balance of opposing brand-specific responses. For example, stronger positive associations with nationally recognized brands could have been nullified by less favorable responses to private labels. Given our design and statistical power, detailed stratification by brand or age group was not feasible. However, future studies should investigate these subgroup differences, as prior work suggests that brand effects are not uniform across consumer segments [1,2].

### 3.2. Emotions and Overall Acceptance

A stepwise regression relating the intensity with which panelists felt each of the emotions to overall acceptance, resulted in an average R-squared value of 0.62 for all soup types combined (Table 1).

Similarly, a second stepwise regression comparing responses for the same key emotions with consumer purchase intent yielded an average R-squared value of 0.67 (Table 2). Thus, we can understand that the change in overall acceptance and purchase intent, respectively, can be explained by changes in the perceptions of the 42 emotions. However, only 3 of the 42 emotions contributed with partial R-squared values greater than 0.01: satisfied, disgusted, and, for the condensed category, bored.

Despite the low partial R-squared values of the other 39 emotions, we wondered if these three emotions were more influential in determining both overall acceptance and purchase intent than our initial analyses revealed. We subsequently performed a pairwise correlation of the hedonic scores of the reported prevalence of each emotion at the time of consumption with the reported prevalence of the emotion “Satisfied” (Table 3). Most of the emotions included in the panel were positive emotions, so we interpreted a higher correlation of each emotion to “Satisfied” to represent a greater influence on both overall acceptance of the product and purchase intent. Using the Emotion Annotation and Representation Language presented by HUMAINE [19], eleven of the thirteen emotions most correlated with the emotion “satisfied” were characterized as “quiet positive” emotions.

Table 4 shows that emotions in this category were characterized as “caring”, “positive thoughts”, “positive and lively”, and “reactive”. “Worried”, the only emotion to have a significant correlation with “disgusted”, was characterized as an emotion that was “negative and not in control”. Although some of these relationships may appear intuitive, their quantified strength and consistency across multiple soup categories demonstrate that confirming and consolidating such effects provides both theoretical clarity and practical guidance for product development and marketing.

A Mixed Models Analysis of Covariance complemented the previous tests, confirming a positive relationship between responses for the emotion “satisfied” and both overall acceptance (Table 5) and purchase intent (Table 6). On average, a 26-point increase in the panelists’ response for this key emotion was associated with a one-point increase in overall acceptance. In contrast, there was an 18-point increase for each 1-point increase in purchase intent. Conversely, the responses to both “disgusted” and “bored” displayed a negative relationship with overall acceptance and purchase intent, albeit with a lesser magnitude. A 31-point increase in the panelists’ response for “disgusted” was associated with a 1-point decrease in overall acceptance, while a 42-point increase for that emotion reflected a 1-point decrease in purchase intent.

Confirmed by the stepwise regressions presented in Table 1 and Table 2, all types of chicken noodle soup displayed a significant relationship between emotional responses and both overall acceptance and purchase intent. Despite yielding the lowest combined R-squared value in both tests, the condensed chicken noodle soup panels maintained that 58% and 67% of the changes in responses for overall acceptance and purchase intent, respectively, could be attributed to changes in the panelists’ responses for the 42 emotions. These values are accepted as statistically significant. Thus, influencing the emotions perceived in the consumption of condensed, chunky, or low-sodium chicken noodle soup would also allow an individual to influence the overall acceptance and purchase intent of a consumer for that soup, perhaps indicating that for this product, intrinsic emotions are more influential than branding.

Because quiet positive emotions have been shown to have the greatest influence on overall acceptance and purchase intent of consumers for condensed, chunky, and low-sodium chicken noodle soup, marketers and product developers representing soup-producing companies should attempt to instill these emotions in consumers at the point of purchase and consumption. Suppose marketers enable consumers to feel content, pleasant, peaceful, secure, and warm at the time of purchase and attribute those emotions to the chicken noodle soup. In that case, consumers will be more likely to purchase the soup than if they had felt any other type of emotion. Additionally, by formulating their soups to evoke the same emotions in consumers at the time of consumption, product developers may increase the acceptance that consumers have for that soup, resulting in a greater likelihood of consumers becoming repeat customers.

A possible application for these results could be in the advertising of comfort foods, which tend to lift the spirits by inducing positive feelings. Several food market reports and studies have discussed consumer preferences for comfort foods [27,28]. The results of the present study suggest, however, that for this advertising to be especially successful, comfort food, such as chicken noodle soup, should especially evoke emotions categorized as “quiet positive emotions.”

Interestingly, “nostalgia” did not have as strong an influence on overall acceptance and purchase intent for chicken noodle soup as the quiet positive emotions. Past marketing campaigns for many popular brands of chicken noodle soup have attempted to evoke feelings of nostalgia among viewers, reminding them of possible fond memories. However, such tactics are unlikely to be the most successful in convincing consumers to purchase chicken noodle soup. Equally less successful is the promotion of interpersonal emotions, whether they are positive and lively or caring. Instead, the more successful marketer will attempt to generate quiet, positive emotions that involve the consumers themselves in the present, rather than focusing on relationships with others or the past. Some companies are already investing in creating new product lines and flavors that could create these quiet positive emotions by observing market trends, especially among millennials and Gen Z as they become a larger share of consumer spending, such as boba tea, canned seafood, and spicy or bold and strong flavors in existing product lines [28,29].

It should be noted that while this study presents findings indicating a relationship between certain perceived emotions and both overall acceptance and purchase intent, it does not review or make any claims about relationships involving the emotions that consumers expect to feel when eating chicken noodle soup at the time of purchase. Rick and Loewenstein [30] explain that when presentations and displays make consumers aware of the emotional pain of opportunity cost associated with forfeiting money in exchange for a good, consumers are less likely to choose to make the transaction. However, research is lacking to verify whether the expectation of positive emotions associated with using a product might induce consumers to complete a purchase. We recommend that subsequent studies account for this disparity by examining how consumers perceive a product and how they anticipate feeling if they choose to consume it. Consumers should be asked to rate their actual emotions after consumption, as well as to indicate any intention to make a repeat purchase.

### 3.3. Limitations

This study has several limitations that should be considered when interpreting the findings. First, the number of samples in each category was uneven due to market availability in the test area. Second, panelists were recruited from a single geographic area, primarily a university community and surrounding neighborhoods. Although the sample included a range of ages and a balanced gender, socioeconomic background, and cultural food practices, prior brand familiarity was not systematically controlled, which may limit generalizability. Third, participants took part in multiple sessions within a soup category, introducing the possibility of carryover effects. Panels were scheduled two weeks apart, and participants were not informed that the sessions were related; however, residual familiarity cannot be entirely ruled out. Fourth the emotion lexicon contained 42 terms, which—while comprehensive—may have been cognitively demanding and included overlapping descriptors (e.g., calm vs. peaceful). Fifth, different scales were used for acceptance (a 9-point hedonic scale) and emotions (a 100-point line scale). This mixed approach reflects common practice in sensory science but may have introduced complexity for participants.

Finally, the statistical analyses were exploratory in nature. Stepwise regression can inflate Type I error; although complementary methods (pairwise correlations, mixed-model ANCOVA) supported the findings, results should be interpreted as associative rather than causal. The post hoc sensitivity power analysis indicated that the study was adequately powered to detect small to moderate effects; however, subtle brand or category effects may have gone undetected. Finally, although brand presence showed no overall effect, we cannot exclude the possibility that positive responses to some brands were offset by negative responses to others, resulting in a net neutral outcome. More granular analyses by brand type or demographic subgroup may be needed to reveal such effects.

## 4. Conclusions

This study highlights that brand presence may have minimal practical influence on consumer acceptance of chicken noodle soup. At the same time, a parsimonious set of emotions, particularly ‘quiet positive’ feelings such as satisfaction, contentment, and warmth, serve as the strongest predictors of purchase intent. By employing a cross-category design and a comprehensive 42-term emotion lexicon, we extend previous sensory research and highlight the importance of linking discrete emotions to both liking and purchasing decisions. These findings suggest that product developers and marketers should prioritize fostering subtle, self-focused positive emotions over brand identity or nostalgia when promoting comfort foods. Future research should employ larger, more diverse samples, refine emotion lexicons, and examine both expected and realized emotions to clarify how emotional anticipation translates into actual consumption and repeat purchasing.

## Figures and Tables

**Table 1:** Stepwise Regression displaying the Partial R-Square between responses for key emotions and overall acceptance for three soup types and combined soup categories. Overall acceptance was measured on a scale where 1 = dislike extremely, 5 = neither like nor dislike, 9 = like extremely. N = 112.

Influence of Pertinent Key Emotions on Overall Hedonic Acceptance				
Emotion	Low-Sodium Soup	Condensed Soup	Chunky Soup	All Soups
Satisfied	0.4995	0.3584	0.4693	0.4515
Disgusted	0.0967	0.1565	0.1564	0.1402
Bored	0.0032	0.0143	0.0026	0.0048
All Emotions	0.6395	0.5805	0.6546	0.6197

**Table 2:** Stepwise Regression displaying the Partial R-Square between responses for key emotions and purchase intent for three soup types and combined soup categories. Purchase intent was measured on a scale where 1 = extremely unlikely, 5 = neither likely nor unlikely, 9 = extremely likely. N = 112.

Influence of Pertinent Key Emotions on Purchase Intent				
Emotion	Low-Sodium Soup	Condensed Soup	Chunky Soup	All Soups
Satisfied	0.5572	0.4946	0.5225	0.5243
Disgusted	0.0237	0.1095	0.1083	0.1004
Bored	0.0807	0.0172	0.0107	0.0152
All Emotions	0.7003	0.6660	0.6738	0.6661

**Table 3:** Pairwise Correlation of R-values. Each was categorized using the Emotion Annotation and Representation Language presented by HUMAINE. “Combined” signifies a composite of the responses for all three panels. For cells shaded in green, R-values > 0.90. For cells shaded blue, R-values > 0.80. For cells shaded pale orange, R-values > 0.70. For cells shaded light gray, R-values > 0.60. * Values for “Worried” give the correlation with “Disgusted”.

Correlation of All Emotions to Response for “Satisfied”					
	Low-Sodium	Condensed	Chunky	Combined	Emotional Group
Content	0.8943	0.8720	0.9076	0.8964	Quiet positive
Pleasant	0.9083	0.8756	0.8790	0.8852	Quiet positive
Good	0.8786	0.8487	0.8831	0.8743	Quiet positive
Relaxed	0.8134	0.8701	0.8950	0.8692	Quiet positive
Peaceful	0.8346	0.8395	0.8731	0.8561	Quiet positive
Glad	0.8538	0.8256	0.8632	0.8524	Quiet positive
Comfortable	0.8384	0.8399	0.8643	0.8523	Quiet positive
Secure	0.8288	0.8456	0.8319	0.8341	Quiet positive
Whole	0.8215	0.7999	0.8201	0.8161	Quiet positive
Good natured	0.8199	0.8135	0.8123	0.8147	Quiet positive
Warm	0.7904	0.7282	0.8449	0.8052	Quiet positive
Calm	0.7004	0.7838	0.7855	0.7639	Quiet positive
Quiet	0.6030	0.6371	0.6431	0.6321	Quiet positive
Mild	0.5516	0.6254	0.6689	0.6314	Quiet positive
Pleased	0.9322	0.8940	0.9323	0.9233	Positive thoughts
Steady	0.7542	0.7818	0.7918	0.7807	Positive thoughts
Nostalgic	0.6648	0.6772	0.7637	0.7170	Positive thoughts
Free	0.6666	0.5939	0.6399	0.6355	Positive thoughts
Friendly	0.7866	0.7245	0.7561	0.7561	Caring
Loving	0.7443	0.7888	0.7395	0.7519	Caring
Understanding	0.7081	0.7238	0.6968	0.7059	Caring
Affectionate	0.7242	0.7005	0.6951	0.7030	Caring
Tender	0.6797	0.6943	0.6938	0.6909	Caring
Interested	0.7605	0.6691	0.7468	0.7286	Reactive
Polite	0.6982	0.6986	0.7091	0.7039	Reactive
Happy	0.8566	0.8297	0.8725	0.8589	Positive and lively
Joyful	0.7803	0.7695	0.7704	0.7721	Positive and lively
Merry	0.7386	0.7187	0.7089	0.7176	Positive and lively
Enthusiastic	0.7369	0.6267	0.7028	0.6911	Positive and lively
Energetic	0.6962	0.5588	0.6751	0.6491	Positive and lively
Active	0.6522	0.5240	0.6446	0.6153	Positive and lively
Eager	0.6109	0.5014	0.5549	0.5539	Positive and lively
Adventurous	0.6168	0.4819	0.6123	0.5793	Unassigned
Daring	0.5019	0.3545	0.3981	0.4109	Unassigned
Aggressive	0.3060	0.1543	0.1419	0.1810	Unassigned
Guilty	0.1221	0.0625	0.0211	0.0516	Negative thoughts
Tame	0.5366	0.4774	0.5985	0.5552	Negative and not in control
Wild	0.3766	0.1628	0.1983	0.2349	Negative and not in control
Worried *	0.6509	0.6534	0.6787	0.6668	Negative and not in control

**Table 4:** Values are the average for all emotions in each emotional group, calculated from the results given in Table 3, except the listed value for “Worried”. For cells shaded blue, the R values range from 0.8 to 0.8999. Pale orange indicates 0.7–0.7999. Light gray indicates 0.6–0.6999.

Average Correlation of Emotions to “Satisfied” by Emotional Group				
	Low-Sodium	Condensed	Chunky	Combined
Quiet positive	0.7955	0.8004	0.8266	0.8131
Positive thoughts	0.7544	0.7367	0.7819	0.7641
Caring	0.7286	0.7264	0.7162	0.7215
Reactive	0.7293	0.6839	0.7280	0.7162
Positive and lively	0.7245	0.6470	0.7042	0.6940
Unassigned	0.4749	0.3302	0.3841	0.3904
Negative thoughts	0.1221	0.0625	0.0211	0.0516
Negative and not in control	0.2711	0.1526	0.2027	0.2075

**Table 5:** Mixed Models Analysis of Covariance yielding the average change in responses to key emotions per one point change in overall acceptance. Emotions are measured on a scale of 0 to 100. Overall acceptance was measured on a 1 to 9 scale where 1 = dislike extremely, 5 = neither like nor dislike, 9 = like extremely.

Incremental Relationship Between Pertinent Emotions and Overall Hedonic Acceptance								
Emotion	Low-Sodium Soup		Condensed Soup		Chunky Soup		All Soups	
	Blind	Not Blind	Blind	Not Blind	Blind	Not Blind	Blind	Not Blind
Satisfied	25.19	25.84	37.17	31.35	24.69	25.45	25.77	26.46
Disgusted	−46.73	−32.36	−32.89	−38.31	−35.09	−29.67	−36.50	−31.15

**Table 6:** Mixed Models Analysis of Covariance yielding the average change in responses to key emotions per one point change in purchase intent. Emotions are measured on a scale of 0 to 100. Purchase intent was measured on a 1 to 9 scale 1 = dislike extremely, 5 = neither like nor dislike, 9 = like extremely.

**Incremental Relationship Between Pertinent Emotions and Purchase Intent**								
Emotion	Low-Sodium Soup		Condensed Soup		Chunky Soup		All Soups	
	Blind	Not Blind	Blind	Not Blind	Blind	Not Blind	Blind	Not Blind
Satisfied	16.64	18.73	20.49	19.34	16.78	17.27	17.48	18.18
Disgusted	−52.36	−44.64	−37.59	−38.91	−46.08	−43.29	−44.84	−41.84
Bored	−63.69	−80.00	−97.09	−69.44	−123.46	−112.36	−93.46	−90.09

## Data Availability

Data available on request due to privacy restrictions.
